# Effect of a Mobile Health Application With Nurse Support on Quality of Life Among Community-Dwelling Older Adults in Hong Kong: A Randomized Clinical Trial

**DOI:** 10.1001/jamanetworkopen.2022.41137

**Published:** 2022-11-09

**Authors:** Arkers Kwan Ching Wong, Frances Kam Yuet Wong, Karen Kit Sum Chow, Siu Man Wong, Jonathan Bayuo, Annie Ka Ying Ho

**Affiliations:** 1 School of Nursing, The Hong Kong Polytechnic University, Hung Hom, Hong Kong; 2 The Hong Kong Lutheran Social Service, Homantin, Hong Kong

## Abstract

**Importance:**

Mobile health (mHealth) smartphone apps are becoming increasingly popular among older adults, although the reactive care approach of these apps has limited their usability.

**Objective:**

To evaluate the effects of an interactive mHealth program supported by a health-social partnership team on quality of life (QOL) among community-dwelling older adults in Hong Kong.

**Design, Setting, and Participants:**

This was a 3-group, randomized clinical trial conducted in 5 community centers in Hong Kong from December 1, 2020, to April 30, 2022, with a last follow-up date of January 31, 2022. Participants included older adults aged at least 60 years who were living within the service area, used a smartphone, and had at least 1 of the following problems: chronic pain, hypertension, or diabetes. Data were analyzed from May 1 to 10, 2022.

**Interventions:**

Participants were randomly assigned to the mHealth with interactivity (mHealth+I) group, mHealth group, or control group. Participants in the mHealth+I group received the mHealth app and nurse case management supported by a health-social partnership team. The mHealth group received the mHealth app only. The control group received no mHealth app or health-social care services.

**Main Outcomes and Measures:**

The primary outcome was the change in QOL from baseline to 3 months after completion of the intervention.

**Results:**

Among 221 participants (mean [SD] age 76.6 [8.0] years; 185 [83.7%] women), 76 were randomized to the control group, 71 were randomized to the mHealth group, and 74 were randomized to the mHealth+I group. The most common chronic diseases or problems were hypertension (147 participants [66.5%]), pain (144 participants [65.2%]), cataracts (72 participants [32.6%]), and diabetes (61 participants [27.6%]). At 3 months after the intervention and compared with the intervention group, there were no statistically significant differences in either the physical component summary (mHealth+I: β = −1.01 [95% CI, −4.13 to 2.11]; *P* = .53; mHealth: β = 0.22 [95% CI, −3.07 to 3.50]; *P* = .90) or the mental component summary (mHealth+I: β = −0.87 [95% CI, −4.42 to 2.69]; *P* = .63; mHealth: β = 1.73 [95% CI, −1.89 to 5.34]; *P* = .35) QOL scores. Only secondary outcomes, including self-efficacy (β = −2.31 [95% CI, −4.26 to −0.36]; *P* = .02), systolic blood pressure (β = −2.30 [95% CI, −5.00 to −0.13]; *P* = .04), pain levels (β = 1.18 [95% CI, 0.52 to 2.00]; *P* = .02), and health services utilization (β = 0.98 [95% CI, 0.32 to 2.09]; *P* = .048) improved in the mHealth+I group compared with the control group.

**Conclusions and Relevance:**

This randomized clinical trial found no difference in the primary outcome between the mHealth+I group and the control group confirming that there were no incremental benefits to adding interactivity in mHealth programs for older adults with chronic diseases.

**Trial Registration:**

Clinicaltrials.gov Identifier: NCT03878212

## Key Points

**Question:**

Can a mobile health (mHealth) smartphone app delivered by a nurse case manager supported by a health-social partnership team improve quality of life among community-dwelling older adults in Hong Kong?

**Findings:**

In this randomized clinical trial with 221 participants, the mHealth program with the proactive support of a nurse case manager and the health-social partnership team was not significantly more effective than either the mHealth program alone or the usual care control in enhancing quality of life among community-dwelling older adults.

**Meaning:**

This randomized clinical trial found that the implementation of an interactive mHealth program with the support of a health-social partnership did not enable older adults to better manage their health in the community.

## Teaser

This randomized clinical trial evaluates the efficacy of a nurse-supported interactive mobile health smartphone app on quality of life among community-dwelling older adults in Hong Kong.

## Introduction

The emergence of mobile health (mHealth) smartphone apps has ushered in an innovative way for older adults to improve their health and prevent deterioration from chronic diseases. To empower older adults with self-care capabilities, mHealth apps generally have several features, including health information delivery, appointment and medication reminders, and monitoring of vital signs.^1,2,3,4^ There is evidence that more older adults nowadays are willing to use their smartphones to better manage their own health.^5,6^ A recent report indicated that introducing mHealth may improve the efficiency of health care for older adults by 20%, suggesting that using a smartphone is a viable way to move the health promotion process forward.^7^

Because increasing numbers of older adults are using smartphones and health-related apps in daily life, it is commonly perceived by those in the mHealth industry that these older users are keen to monitor their health via technology and are capable and informed enough to interpret health information and know when to seek help from health care professionals.^8^ However, the evidence indicates otherwise—most older adults tend not to send messages to health care professionals even when experiencing severe symptoms, for fear of disturbing people.^8^ It is worth acknowledging that mHealth apps, although they serve as an interactive platform for older adults and health care professionals, may not necessarily promote patient-clinician communication. Studies suggest that nurses failed to review the health conditions of older adults on a daily basis because of time constraints.^4,8,9^ They also regard the regular tracking of data as unnecessary because they rely on alerts triggered when readings outside of reference ranges are reported.^8^ Insufficient interaction with health care practitioners could provoke uncertainty and anxiety among older adults, especially when they encounter difficulties in managing their own health.^3^

Commonly appointed as part of a group of mHealth administrators, nurses are highly competent to provide health education, modify health beliefs, and manage chronic health symptoms for older adults.^9^ When nurses encounter problems beyond their scope of practice, such as depressive symptoms and acute illnesses as reported in the app, they can seek support from a health-social partnership team that includes social workers and general practitioners to provide seamless and coordinated care. Previous studies indicate that older adults who received integrated care supported by a health-social partnership team experienced a significant improvement in their QOL, activities of daily living, and depressive symptoms,^10^ although the program was not delivered through an mHealth app. A comprehensive understanding of health and social conditions, as achieved through interprofessional collaboration, is a prerequisite for achieving continuity of care for older adults.^11^ To our knowledge, there are no studies on supporting an mHealth program with an integrated health-social partnership team when further help is needed by older adults. Therefore, this study focuses on the implementation of a proactive mHealth app to promote self-care ability and health among older adults. The differential benefits of adding nurse interactions supported by an integrated health-social partnership model in the use of mHealth is examined.

The study’s aim was to test the effectiveness of an mHealth with interactivity program on self-management outcomes (ie, QOL, self-efficacy, blood pressure, capillary blood glucose level, pain score, and depression), and health service utilization outcomes (ie, outpatient clinic visits, emergency department visits, and hospital admissions).

## Methods

This randomized clinical trial was approved by the Human Subjects Ethics Subcommittee of the Hong Kong Polytechnic University. All participants were provided with a full explanation of the study and signed informed consent forms prior to baseline data collection. The study followed the Consolidated Standards of Reporting Trials (CONSORT) reporting guideline for randomized clinical trials. The trial protocol and statistical analysis plan are provided in Supplement 1.

### Design and Settings

A single-blinded, 3-group randomized clinical trial design was adopted in this study. The participants were recruited from 5 community and elderly centers serving more than 50 000 older adults per year.

### Participants, Recruitment Strategy, and Randomization

Our previous study showed that pain, hypertension, and diabetes are the most prevalent health problems among community-dwelling older adults.^12^ Therefore, we recruited older adults with at least 1 of these conditions who also fulfilled the following criteria: (1) aged 60 years or older, (2) living within the service areas, and (3) a smartphone user. The participants were ineligible if they were: (1) already participating in other mHealth programs, (2) experiencing diagnosed psychiatric problems, (3) bed-bound, or (4) had no internet coverage.

From December 1, 2020, to April 30, 2022, the community center staff called potentially eligible participants from their membership list, confirmed their eligibility, explained the program to them, and sought their consent to participate. In the center, a research assistant (RA) collected the baseline data of those who agreed to participate, and called the principal investigator (A.K.C.W.) to randomize the participants. The principal investigator generated a set of random numbers (ie, 1 = mHealth with interactivity [mHealth+I] group; 2 = mHealth group; 3 = control group) using Research Randomizer software (Social Psychology Network), placed them in sealed envelopes, and sequentially revealed them when he received the call from the RA. Both the RA and community center staff were blinded to the group assignments.

### Sample Size

The sample size was calculated based on a power analysis.^13^ Assuming a power of 80%, a level of significance of 5%, and a Cohen *d* effect size of 0.2 from a previous similar study with the same primary outcome (QOL),^12^ the required sample size was 60 per group. Assuming a 20% dropout rate,^12^ the total sample size needed was 72 participants per group, ie, a total of 216 participants.

### Interventions

Three groups were involved in this study: the mHealth+I group, the mHealth group, and the control group. Details of each group are provided in our trial protocol in Supplement 1.^14^

#### mHealth With Interactivity Group

Each participant in the mHealth+I group received 2 components of the intervention: (1) an mHealth smartphone app designed by the research team with technical support from one of the biggest telecommunications companies in Hong Kong, and (2) a nurse case management model in partnership with a health-social care team composed of social workers and general practitioners.

The intervention’s 2 components were guided by the 3 levels of Bronfenbrenner’s Ecological Systems Theory: microsystem, mesosystem, and macrosystem.^15^ At the microsystem level, participants were encouraged to input the following information into the mHealth app: their daily vital signs, such as blood pressure, capillary glucose level, and heart rate, and the presentation of symptoms, such as dizziness, pain, and vomiting. A nurse would review the participant’s entry daily. The working protocols for each problem involved 3 levels: nurse advice on self-management, coordination with social workers or general practitioners, and referral to the next level of care (eg, a hospital). They were developed according to the Omaha System and the National Institute for Health and Care Excellence guidelines.^16,17^

At the mesosystem level, the nurse made 8 proactive calls to each participant in the mHealth+I group over the 3-month program period (ie, first month: a weekly call; second and third months: a biweekly call). During these calls, the nurse provided a comprehensive health assessment, and assisted and empowered the participants to set contract goals, identified facilitators and barriers to achieving the goals, and provided individualized education on self-care health management. To increase the self-efficacy level of the participants toward self-management, interventions guided by the Bandura self-efficacy theory were given, such as recalling previous effective self-management strategies, noting the beneficial effects of adopting self-management behavior, and verbal encouragement.^18^

At the macrosystem level, a coordinated health and social partnership was built based on Gittell’s relational coordination theory.^19^ A biweekly case conference was held among the nurse, social workers, and general practitioners to discuss the participant’s progress and current condition, suggest revisions or modifications of goals, and address the participants’ concerns. They also jointly developed a set of standardized interventions and referral protocols to identify and understand each other’s caring roles.

#### mHealth Group

The mHealth group only received functions provided in the mHealth app. They received no support from a nurse case manager or from members of the health-social partnership team. However, the nurse would call the participants if they reported items of concern in the app.

#### Control Group

The control group received no mHealth app or health-social care services. Similar to the other 2 groups, the participants in this group could attend events organized by the community centers, such as health talks and recreational activities.

### Data Collection

Data were collected at 2 time points: baseline and 3 months after the intervention. The RA, who was trained and blinded to the group assignment, was responsible for collecting the data through a face-to-face survey in the community centers.

### Outcome Measures

There were 2 sets of outcome measures: self-management and health service utilization outcomes. Demographic data on age, gender, level of education, and comorbidity were also collected at baseline.

#### Self-management Outcomes

The primary outcome of this study was QOL. Although self-efficacy was prespecified as the primary outcome in the trial protocol (Supplement 1), QOL is a more meaningful clinical parameter for the end users. The outcome switch occurred prior to the trial commencement informed by QOL as the primary outcome. For example, the sample size calculation was performed based on the QOL. QOL was measured by using the 12-item Short Form Health Survey version 2–Chinese (HK) version.^20^ Scores are summarized into the Physical Component Summary (PCS) and Mental Component Summary (MCS), with higher scores indicating better QOL.^20^

Self-efficacy was measured using the 10-item, 4-point Likert General Self-efficacy Scale. Scores range from 10 to 40, with higher scores representing better self-efficacy. The scale has been widely validated with high reliability.^21^

Blood pressure was measured with a calibrated electronic sphygmomanometer, and capillary blood glucose levels were measured by a capillary glucose meter. The results on capillary blood glucose levels were divided in a dichotomous format (outside vs within reference range). Based on World Health Organization guidelines,^22^ capillary blood glucose levels of between 70.27 mg/dL and 100.90 mg/dL (to convert to millimoles per liter, multiply by 0.0555) were regarded as within reference range.

The pain score was extracted from the visual analogue scale installed in the app. Scores ranged from 0 to 10, with lower scores representing lower levels of pain.

Depression was evaluated using the Geriatric Depression Scale, a 15-item Likert scale with scores ranging from 0 to 15. Higher scores indicate more severe depressive symptoms.^23^

#### Health Service Utilization Outcomes

Health service utilization was measured by the number of times the participant visited a general practitioner clinic, government out-patient clinic, emergency department, or hospital. The information was collected by subjective reports from participants and confirmed with medical and attendance certificates, with good reliability.^12^

### Statistical Analysis

Statistical tests were conducted using the SPSS statistical software version 26 (IBM). Baseline demographic data were presented using count numbers and percentages. The outcomes for each group were presented in terms of mean and SE. The generalized estimating equation was adopted to identify the between-group, within-group, and interaction effects between time and group. The exchangeable working correlation matrix was used to emphasize the same spacing between repeated measurements for each participant. Intention-to-treat was used for the primary analysis. A result was significant at 2-tailed *P* < .05. Data were analyzed from May 1 to 10, 2022.

## Results

### Baseline Characteristics

Among 221 participants (mean [SD] age 76.56 [7.96] years; 185 [83.7%] women) included, 76 were randomized to the control group, 71 were randomized to the mHealth group, and 74 were randomized to the mHealth+I group. Most participants had a primary education or above (171 participants [77.4%]). The 4 most common chronic diseases or problems that the participants experienced were hypertension (147 participants [66.5%]), pain (144 participants [65.2%]), cataracts (72 participants [32.6%]), and diabetes (61 participants [27.6%]). There were no statistically significant differences in either participant characteristics or outcome measures at baseline between the groups. The participant recruitment flowchart is presented in the Figure, and Table 1 shows the demographic characteristics of the participants at baseline.

**Figure.**
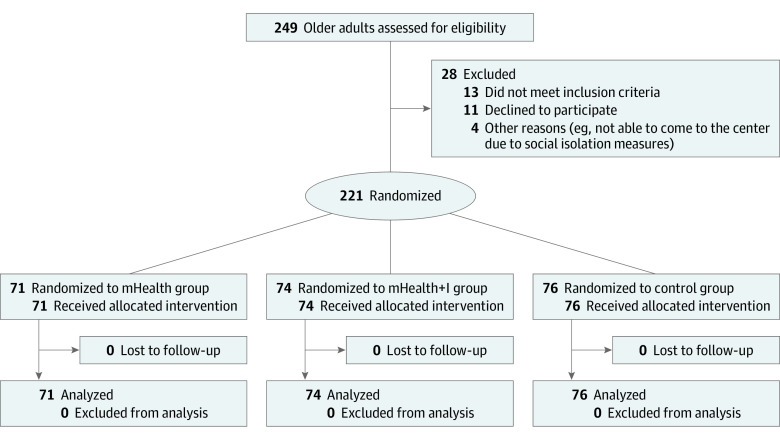
Participant Recruitment Flowchart mHealth+I indicates mobile health app plus interactive support from a nurse case manager supported by a health-social partnership team.

**Table 1.** Demographic Characteristics of the Participants

Characteristic	Group count, No. (%)			
	Total (N = 221)	mHealth+I (n = 74)	mHealth (n = 71)	Control (n = 76)
Gender				
Men	36 (16.3)	14 (18.9)	9 (12.7)	13 (17.1)
Women	185 (83.7)	60 (81.1)	62 (87.3)	63 (82.9)
Age, y				
Mean (SD)	76.6 (8.0)	74.7 (7.6)	77.6 (7.84)	77.4 (8.2)
Median (range)	76 (60-98)	73.5 (60-98)	78 (60-91)	77.5 (63-95)
Education				
No formal education	50 (22.6)	16 (21.6)	20 (28.20)	14 (18.4)
Primary	96 (43.4)	32 (43.2)	29 (40.8)	35 (46.1)
Secondary	69 (31.2)	25 (33.8)	19 (26.8)	25 (32.9)
Tertiary or above	6 (2.7)	1 (1.4)	3 (4.2)	2 (2.6)
Comorbidity^a^				
Pain	144 (65.2)	50 (67.6)	51 (71.8)	43 (56.6)
COPD	11 (5.0)	2 (2.7)	4 (5.6)	5 (6.6)
Hypertension	147 (66.5)	44 (59.5)	46 (64.8)	57 (75.0)
Diabetes	61 (27.6)	22 (29.7)	23 (32.4)	16 (21.1)
Genital diseases	13 (5.9)	8 (10.8)	1 (1.4)	4 (5.3)
Stroke	17 (7.7)	5 (6.8)	5 (7.0)	7 (9.2)
Cancer	22 (10.0)	6 (8.1)	9 (12.7)	7 (9.2)
Arthritis	45 (20.4)	15 (20.3)	15 (21.1)	15 (19.7)
Depression	11 (5.0)	5 (6.8)	3 (4.2)	3 (3.9)
CHD	17 (7.7)	6 (8.1)	5 (7.0)	6 (7.9)
IHD	6 (2.7)	1 (1.4)	1 (1.4)	4 (5.3)
Fracture	18 (8.1)	9 (12.2)	5 (7.0)	4 (5.3)
Cataract	72 (32.6)	25 (33.8)	22 (31.0)	25 (32.9)

Abbreviations: CHD, congestive heart disease; COPD, chronic obstructive pulmonary disease; IHD, ischemic heart disease; mHealth, mobile health; mHealth+I, mHeath app plus support from a nurse case manager supported by a health-social partnership team.

^a^ The participants had to report chronic diseases that were diagnosed by a certified doctor and could be shown in the Clinical Management System.

### Self-management Outcomes

#### Primary Outcome: QOL

All 3 groups had better PCS scores at 3 months compared with baseline (Table 2). However, at 3 months after the intervention and compared with the control group, there were no statistically significant differences in either the PCS (mHealth+I: β = −1.01 [95% CI, −4.13 to 2.11]; *P* = .53; mHealth: β = 0.22 [95% CI, −3.07 to 3.50]; *P* = 90) or the MCS (mHealth+I: β = −0.87 [95% CI, −4.42 to 2.69]; *P* = .63; mHealth: β = 1.73 [95% CI, −1.89 to 5.34]; *P* = .35) QOL scores. Similarly, there were no statistically significant within-group or interaction effects between group and time in both PCS and MCS scores in all 3 groups (Table 3).

**Table 2.** Effectiveness Outcomes At 2 Different Time Points

Outcomes	Group, mean (SE)		
	Control	mHealth	mHealth+I
Quality of life			
PCS score			
T2	41.62 (0.93)	42.85 (1.25)	42.14 (1.04)
T1	39.76 (1.17)	39.98 (1.20)	38.75 (1.08)
MCS score			
T2	49.67 (1.25)	50.4 (1.20)	49.51 (1.09)
T1	50.58 (1.32)	52.31 (1.29)	49.71 (1.25)
Self-efficacy score			
T2	26.28 (0.61)	27.73 (0.62)	26.78 (0.73)
T1	26.70 (0.73)	26.15 (0.81)	24.39 (0.68)
Blood pressure			
Systolic			
T2	137.81 (3.05)	137.13 (2.01)	132.95 (2.34)
T1	136.83 (2.73)	139.51 (0.81)	136.27 (2.09)
Diastolic			
T2	71.44 (1.56)	71.74 (1.79)	70.41 (1.58)
T1	71.72 (1.24)	71.99 (1.22)	71.02 (1.16)
Pain level			
T2	3.14 (2.49)	3.12 (2.01)	2.90 (1.52)
T1	3.28 (2.73)	3.2 (2.49)	3.30 (2.09)
Depression score			
T2	3.75 (0.35)	3.54 (0.37)	3.81 (0.33)
T1	3.63 (0.37)	4.08 (0.44)	4.47 (0.41)
Unplanned health care utilization, No.			
Total			
T2	0.47 (0.11)	0.30 (0.11)	0.64 (0.21)
T1	1.88 (0.36)	2.49 (0.55)	2.77 (0.50)
Visits			
GOPD			
T2	0.27 (0.13)	0.03 (0.03)	0.26 (0.06)
T1	0.29 (0.09)	0.31 (0.15)	0.93 (0.32)
General practitioner			
T2	0.33 (0.10)	0.24 (0.11)	0.45 (0.15)
T1	1.05 (0.22)	0.9 (0.21)	1.23 (0.25)
ED			
T2	0.04 (0.02)	0.03 (0.02)	0.01 (0.01)
T1	0.16 (0.05)	0.17 (0.06)	0.11 (0.04)
Hospital admission			
T2	0.01 (0.01)	0 (0)	0.03 (0.02)
T1	0.09 (0.04)	0.18 (0.09)	0.08 (0.03)

Abbreviations: GOPD, governmental out-patient department; mHealth, mobile health; mHealth+I, mHealth app plus support from a nurse case manager supported by a health-social partnership team; MCS, mental component summary; PCS, physical component summary; T1, baseline; T2, immediate postintervention follow-up.

**Table 3.** Parameter Estimates of Effectiveness Outcomes

Measure	β (95% CI) [*P* value]						
	Intercept	Group effect		T2	Interaction effect	
		mHealth+I group	mHealth group		mHealth+I Group	mHealth Group
Quality of life						
PCS	39.76 (37.47 to 42.06) [<.001]	−1.01 (−4.13 to 2.11) [.53]	0.22 (−3.07 to 3.50) [.90]	1.86 (−0.08 to 3.80) [.06]	1.53 (−1.45 to 4.52) [.31]	1.02 (−2.44 to 4.48) [.56]
MCS	50.58 (48.00 to 53.16) [<.001]	−0.87 (−4.42 to 2.69) [.63]	1.73 (−1.89 to 5.34) [.35]	−0.91 (−2.92 to 1.10) [.37]	0.71 (−2.48 to 3.90) [.66]	−0.99 (−4.17 to 2.19) [.54]
Self−efficacy	26.70 (25.78 to 28.12) [<.001]	−2.31 (−4.26 to −0.36) [.02]	−0.54 (−2.68 to 1.59) [.62]	−0.42 (−2.02 to 1.17) [.61]	2.81 (0.74 to 4.89) [.008]	2.00 (−0.135 to 4.13) [.07]
Blood pressure						
Systolic	136.83 (133.37 to 140.30) [<.001]	−2.30 (−4.25 to −0.35) [.04]	2.68 (−2.57 to 7.92) [.31]	0.98 (−4.81 to 6.76) [.74]	−2.48 (−4.00 to −0.96) [.03]	−3.36 (−11.37 to 4.66) [.41]
Diastolic	71.72 (69.30 to 74.15) [<.001]	−0.70 (−4.02 to 2.63) [.68]	0.26 (−3.15 to 3.68) [.88]	−0.28 (−3.20 to 2.64) [.85]	−0.34 (−4.50 to 3.83) [.87]	0.04 (−4.90 to 5.00) [.99]
Capillary blood glucose level	−1.78 (−2.42 to −1.14) [<.001]	−0.33 (−1.31 to 0.64) [.50]	0.28 (−0.60 to 1.16) [.53]	−0.49 (−1.77 to 0.79) [.45]	0.77 (−1.06 to 2.59)	−0.13 (−1.86 to 1.59) [.88]
Pain level	1.98 (0.36 to 2.34) [<.001]	1.18 (0.52 to 2.00) [.02]	−0.21 (−3.10 to 2.68) [.72]	−0.11 (−0.21 to −0.03) [.01]	−2.24 (−4.01 to −0.47) [.01]	−0.01 (−0.65 to 0.53) [.79]
Depression	3.63 (2.90 to 4.36) [<.001]	0.84 (−0.25 to 1.93) [.13]	0.45 (−0.68 to 1.59) [.43]	0.12 (−0.57 to 0.81) [.74]	−0.78 (−1.71 to 0.145) [.10]	−0.67 (−1.72 to 0.38) [.21]
Unplanned health care utilization						
Total No.	1.88 (1.18 to 2.59) [<.001]	0.98 (0.32 to 2.09) [.048]	0.61 (−1.01 to 2.24) [.46]	−1.41 (−2.15 to−0.67) [.001]	−0.53 (−1.80 to 0.75) [.42]	−0.79 (−2.41 to 0.83) [.34]
Visits						
GOPD	0.29 (0.11 to 0.47) [.001]	0.68 (0.01 to 1.30) [.048]	0.02 (−0.33 to 0.37) [.91]	−0.22 (−0.40 to −0.05) [.01]	−0.65 (−1.13 to −0.17) [.049]	−0.06 (−0.40 to 0.29) [.74]
General Practitioner	1.05 (0.63 to 1.47) [<.001]	0.18 (−0.46 to 0.82) [.59]	−0.15 (−0.74 to 0.44) [.61]	−0.72 (−1.19 to −0.26) [.002]	−0.06 (−0.74 to 0.62) [.86]	0.06 (−0.53 to 0.66) [.84]
ED	0.16 (0.06 to 0.26) [<.001]	−0.05 (−0.17 to 0.07) [.42]	0.01 (−0.14 to 0.16) [.89]	−0.12 (−0.21 to −0.03) [.009]	0.02 (−0.09 to 0.14) [.69]	−0.02 (−0.17 to 0.13) [.77]
Hospital admissions	0.09 (0.02 to 0.17) [.02]	−0.01 (−0.11 to 0.09) [.82]	0.09 (−0.10 to 0.29) [.36]	−0.08 (−0.16 to 0.00) [.05]	0.03 (−0.08 to 0.13) [.65]	−0.10 (−0.30 to 0.09) [.30]

Abbreviations: ED, emergency department; GOPD, governmental out-patient department; mHealth, mobile health; mHealth+I, mHealth app plus support from a nurse case manager supported by a health-social partnership team; MCS, mental component summary; PCS, physical component summary.

#### Secondary Outcomes

##### Self-efficacy

The mHealth+I group had statistically significant lower self-efficacy scores (β = −2.31 [95% CI, −4.26 to −0.36]; *P* = .02) compared with the control group (Table 3). There was no time effect in self-efficacy level in all 3 groups (β = −0.42 [95% CI, −2.02 to 1.17]; *P* = .61). In interaction analysis, the mHealth+I group at 3 months had statistically significant higher self-efficacy scores (β = 2.81 [95% CI, 0.74 to 4.89]; *P* = .008) than the control group at baseline.

##### Systolic and Diastolic Blood Pressure

As with self-efficacy levels, the mHealth+I group had statistically significant better systolic blood pressure (β = −2.30 [95% CI, −4.25 to −0.35]; *P* = .04) than the control group. The interaction effect between the mHealth+I group at 3 months and the control group at baseline was also statistically significant (β = −2.48 [95% CI, −4.00 to −0.96]; *P* = .03).There were no statistically significant group, time, or interaction effects in diastolic blood pressure for all 3 groups at baseline and 3 months (Table 2).

##### Capillary Blood Glucose Levels

We did not calculate the mean for capillary blood glucose levels because the result was divided into 2 categories: within reference range or hyperglycemia. Based on the parameter estimates shown in Table 3, there were no between-group, within-group, or interaction effects found on the capillary blood glucose levels.

##### Pain Levels and Depression

The mHealth+I group had statistically significantly better pain levels (β = 1.18 [95% CI, 0.52 to 2.00]; *P* = .02) and in the time interaction analysis (β = −0.11 [95% CI, −0.21 to −0.03]; *P* = .01). Interaction effects on pain levels were found between the mHealth+I group at 3 months and control group at baseline (β = −2.24 [95% CI, −4.01 to −0.47];, *P* = .01) (Table 3).There were no statistically significant group, time, or interaction differences in depression scores (Table 3).

##### Health Service Utilization Outcomes

In general, all 3 groups showed a statistically lower numbers of health service utilizations at 3 months compared with baseline. The mHealth+I group, in particular, had a statistically significant lower number of unplanned visits to the governmental outpatient department (GOPD) (β = 0.68 [95% CI, 0.01 to 1.30]; *P* = .048) and total number of unplanned health service utilizations (β = 0.98 [95% CI, 0.32 to 2.09]; *P* = .048) than the control group (Table 3). Statistically significant interaction effects between the mHealth+I group at 3 months and control group at baseline were observed regarding unplanned GOPD visits (Table 3).

## Discussion

This randomized clinical trial is one of the first studies to our knowledge to use a proactive nurse case management model with the support of a health-social partnership team to help older adults integrate a disease self-management app in their daily life. mHealth apps are now commonly used to promote health self-management among older adults with chronic diseases. However, the lack of guidance given to older adults on how to use the multiple features of these apps may result in little benefit. The nurse case manager in the mHealth+I program not only encouraged the older adults to use the app by demonstrating and explaining its functions, but also by empowering them to manage their symptoms and equipping them with sufficient skills, knowledge, and confidence to lead a relatively independent life at home.

Compared with the control group, we found no statistically significant finding regarding our primary outcome, QOL. The negative finding particularly in the mHealth+I group is noteworthy considering its comprehensive nature. In the mHealth+I group, the nurse was well-positioned to act as the case manager and played a major role in promoting use of the app, monitoring and retrieving valid and vital health information from the app database, and providing individualized comments to the participants during the proactive telephone calls. General practitioners provided professional medical advice and treatment, while social workers were responsible for meeting the psychosocial needs of the participants and mobilizing community resources. Through codeveloping the working protocols, sharing the participants’ health profiles in the app database, and holding regular team case conferences, the team members were familiar with each other’s interventions, enabling them to provide synergized and integrated care to meet the complex health and social needs of the older adults and complement the effects of the app. Together, these strategies may have contributed to the timely identification and resolution of emerging health problems. However, the frequent and fixed number of contacts with the health-social team members may also have interfered with the basic routine work and social life of those older adults without acute problems, thus reducing their QOL. On the other hand, for older adults with acute problems, the use of phone calls without face-to-face interactions may have limited their ability to indicate the exact location of the problem to the health-social team members. The health-social team members may also have been unable to observe nonverbal cues, perform in-depth physical examinations, conduct imaging tests and blood work, or make diagnoses requiring a more hands-on approach. In future studies, the use of flexible telecare meetings between older adults and a health-social team can be explored to improve the QOL of community-dwelling older adults, especially when face-to-face meetings are not possible.

Having an experienced nurse to guide every step of the program, from contributing to the development of the app, instructing and engaging the participants in its use, and monitoring the health of the participants, to leading a high-functioning health-social partnership team might have contributed to the statistically significant interaction effects in self-efficacy, systolic blood pressure, pain level, and unplanned GOPD visits between the mHealth+I and the control groups. Most health promotion mobile apps lack a theoretical foundation and do not meet the needs and preferences of older adults with chronic diseases.^24^ Equipped with extensive clinical and community care experience, the nurse in this study was able to incorporate evidence-based clinical best practices and medical guidelines into our app and ensure that the information provided, including the medications and treatment choices, would not potentially harm the older adults. Involving the nurse in the creation of our app also helped to instill trust in the participants regarding the credibility and reliability of the contents of the app and to precisely tailor health and medical information to the needs and level of understanding of the older adults. These features were intended to increase the usability of the app, help the older adults to receive the best care decisions, and maintain their motivation and intention to use the app.

### Limitations

The study had several limitations. First, the results are not generalizable to older adults with no smartphone or internet coverage at home. Second, the participants in both intervention groups received help from the nurse only during office hours. Third, the participants’ experiences with using the app were not explored.

## Conclusions

This randomized clinical trial tested the incremental benefits of adding interactivity in the mHealth program, but no significant result was found in the primary outcome, QOL. While mHealth apps contain a variety of functions that enable users to support their health, many existing apps do not proactively provide a direct communication channel between users and health care professionals until users report vital signs outside of reference ranges. Future studies may consider integrating an artificial intelligence decision support system in the app to facilitate disease management for older adults.

## Notes

Supplement 1.
Trial Protocol and Statistical Analysis Plan

Supplement 2.
Data Sharing Statement
