# Supplementary material for: Effect of a Mobile Health Application With Nurse Support on Quality of Life Among Community-Dwelling Older Adults in Hong Kong: A Randomized Clinical Trial
Source: JAMA Netw Open. 2022 Nov 9;5(11):e2241137. doi: 10.1001/jamanetworkopen.2022.41137 (PMC9647479; doi:10.1001/jamanetworkopen.2022.41137)
Supplement: Supplement 1. — Trial Protocol and Statistical Analysis Plan [file jamanetwopen-e2241137-s001.pdf]

Methods Article

A proactive mobile health application program for promoting self-care health management among older adults in the community: study protocol of a three-arm randomized controlled trial

Arkers Kwan Ching WONG[1], Frances Kam Yuet WONG[1], Katherine Ka Pik CHANG[1]

[1] School of Nursing, The Hong Kong Polytechnic University, Hung Hom, Hong Kong

Abstract

Background: The use of mobile health (mHealth) has become common in recent years and is regarded as one of the most effective interventions for developing disease-specific management skills and establishing confidence in making preventive health behavior changes and accomplishing health-related goals among community-dwelling older adults. Most mHealth designs adopt a reactive care approach whereby healthcare professionals do not respond until they receive abnormal assessment results from the database or a message or signal from the client. The purpose of this study is to determine the effectiveness of a proactive mobile health application program with the support of a community health-social care team for older adults dwelling in the community on improving their self-care health management.

Methods: This is a three-arm, randomized controlled trial. The study will be conducted in seven community centers with an estimated sample size of 282 participants. The participants will be randomly assigned to mHealth with interactivity, mHealth, and control groups when they are (1) aged 60 or above, (2) complaining chiefly of pain, hypertension, or diabetes mellitus, (3) living within the service areas, and (4) smartphone users. Subjects in the mHealth with interactivty group will receive two main elements, the mHealth application and nurse case management supported by a social service team. The mHealth group will receive the mHealth application only. The primary outcome measure will be self-efficacy, and secondary outcomes will include self-management outcomes (pain score, blood pressure, capillary blood glucose), client outcomes (quality of life, depression), and health service utilization outcomes (institutionalization and health service utilization

[general practitioner, outpatient clinic, emergency room, hospital admission]). Data will be collected pre-intervention (T1), post-intervention (T2), and three months post-intervention (T3).

Discussion: The incremental benefits of adding interactivity in the mHealth program have not been confirmed. This present study will add valuable information to the knowledge gap of whether mHealth with nurse interaction supported by a health-social partnership can improve self-care management among community-dwelling older adults.

Introduction

The use of mHealth has enjoyed increasing popularity among older adults due to the high penetration rate of mobile phones. According to the Pew Research Center, more than 96% of American adults currently own a smartphone, and the fastest-growing demographic is people over 65, of whom 53% now own a device, representing an 11% increase compared to two years ago (2017) [1]. A similar situation can be found in Hong Kong, where more than 60% of older adults are now using smartphones [2]. There is also emerging evidence that a growing number of older adults have shown a positive attitude towards using smartphones to better manage their health in daily life [3]. The European Commission estimates that the efficiency of providing health care services for older adults will improve by 20% just by introducing mHealth [4], which suggests that employing the smartphone as a platform for promoting health may be a viable way forward.

The World Health Organization (WHO) defined mHealth as a medical and public health practice using the core utility of mobile and wireless technologies to support the achievement of health objectives [5]. mHealth applications (apps) contain sophisticated features that enable users to develop disease-specific management skills, establish confidence in making preventive health behavior changes, and accomplish health-related goals. A systematic review has shown that the features varied, including compositions of assessment, monitoring and documenting symptom severity, chief complaints, and treatment preferences that were reported by older adults through questionnaires, provision of health information via videos, written learning content from credible sources, and delivery of algorithm-based, individually tailored feedback based on the assessment results [6]. Apps also offer a range of interactive possibilities to allow older adults to receive instant support from healthcare professionals by pressing a help button or sending a text message to the system [7]. Healthcare professionals have access to the database via a mobile phone, whereby they receive alerts from the older adults that prompt them to take timely and appropriate action.

Although mHealth has a variety of functions as mentioned, many of the existing apps adopt a reactive care approach, whereby health care professionals do not provide individual care or meet the specific needs of older adults until they receive abnormal assessment results from the apps database or request a message or signal from an older adult. A randomized controlled trial was conducted using an app to increase self-management among older adults with chronic obstructive pulmonary disease [8]. The app included a tool to enable patients to report personal health status, and an interactive platform to enable nurses to develop plans with patients, and give instructions on self-management according to their reported health status and problems. Results showed that the

program had no beneficial impact on the health status and self-efficacy of patients with nurse support as compared to access to online self-management information alone. Another literature review showed that some older adults achieved a sense of control and independence over their daily lives after using mobile apps that included provision of self-care information through a hyperlink to a website, messages and reminders tailored to the individual, and a default system that alerted nurses when their clients had emergency or critical health status [9]. Interestingly, the subjects were found to have greater feelings of loneliness, poor social functioning and poor overall psychological well-being [9]. The developers of these apps often assume that older adults are enthusiastic about monitoring their health via technology, that what they have filled out in assessment tools through the apps are objective facts, and that they have no problem understanding and interpreting the information [10]. Evidence suggests that older adults may not take the initiative in giving information or making contact with health care professionals via the apps due to their lack of ability to judge the appropriate time to request service, or their lack of adequate knowledge to fully express their symptoms or chief complaints in the apps [11]. Older adults in one qualitative study indicated that although most mobile apps contain a help button to facilitate their contact with healthcare professionals at any time, they chose not to use this function even when suffering from severe health problems, as they did not want to disturb the healthcare providers [12]. One systematic review suggested that the reactive care approach does not guarantee quality of communication [13]. Nurses who are responsible for providing care via apps in one study mentioned that they relied on the alerts received when older adults reported severe health concerns. They did not log on to the system daily to check the health conditions of the older adults, as the system would generate alerts if severe health concerns were reported by the users [12]. When apps-mediated communication is not reciprocal, the use of apps may lose its ability to meet the individual needs of older adults, which may subsequently lead to poor quality of life and an increase in health service utilization.

Older adults generally have multiple chronic conditions and physical and psychosocial needs that mean they would benefit from an integrated health-social team to support their independent living in the community. As nurses are equipped with multiple skills, such as judgment and decision-making skills, they can play a pivotal role in healthcare education and symptom monitoring and management for older adults in many of the mHealth applications [14]. When nurses need help in solving problems that are beyond the scope of their professional practice, a referral system that is installed in the app can help them to seek advice from other healthcare professionals and offer seamless care to their clients [17]. Due to their busy schedule, nurses are sometimes unable to follow up on cases, and they seldom communicate with specialists after referring patients to them [15]. In such cases,

communication among professionals is hampered, the care tends to be fragmented, and the results often lead to clients' physical and psychosocial needs remaining unmet [15]. A report revealed that for older adults who have diabetes and chronic obstructive pulmonary disease, nurses only exchange information with other healthcare professionals through referral forms that are provided in the program [16]. Another source also pointed out is that there are no community care services to support the operation of existing apps, the extent of the services nurses can provide is limited [17]. Collaboration and communication between the multidisciplinary team and older adults is therefore fundamental to support independent living, for which effective collaboration among the professionals is a precondition [15]. It is thus crucial to provide a structure of relational and communication links among the health-social team in an mHealth program, with the aim of aligning interventions and providing continuity of care to older adults. This study therefore endeavors to develop a proactive mHealth application to promote self-care ability and health among older adults, examining the differential benefits of adding nurse interaction supported by an integrated health-social partnership model in the use of mHealth. It is hypothesized that participants who receive a proactive mHealth application with the support of health-social partnership will have statistically significant better outcomes than the other two groups; while participants who receive an mHealth application will provide statistically significant better outcomes than the participants who receive the usual care. This study can add to the knowledge gap in the question of whether mHealth with nurse interaction supported by a health-social partnership can improve quality of life and self-care among older adults dwelling in the community.

Methods/Design

The SPIRIT statement was used as a guideline for this protocol paper [18].

Study design and setting

This is a single-blinded, three-armed randomized controlled trial. The research assistant who collects the data is blinded, but the subjects and health and social care providers who are involved in the intervention are not. This study is being conducted in collaboration with a leading local telecommunications company, community centers, and district counsellors. Seven community centers that serve more than 50,000 older adults under these organizations will be used to recruit subjects to maximize the generalizability effect of this program. The present study is conducted according to the principles indicated in the Declaration of Helsinki and is registered at ClinicalTrials.gov (NCT03878212).

Participants and recruitment strategy, and randomization

Our previous study [17] has identified that pain, hypertension, and diabetes mellitus are the most prevalent health problems among older adults living in the community. We are therefore targeting community-dwelling older adults with at least one of these problems but with the ability to self-care. The inclusion criteria of this program are: (1) being aged 60 or above, (2) suffering chiefly from pain, hypertension, or diabetes mellitus, (3) living within the service areas, and (4) being a smartphone user. The participants were not eligible if they were: (1) already engaged in other mHealth programs, (2) diagnosed with psychiatric problems, (3) bed-bound, or (4) living in an area with no Internet coverage.

Identification of individuals that meet the inclusion criteria will be facilitated by the membership lists of the community centers. Center staff will first randomize the number in the membership list, and then call these members. All center staff members who are involved in recruitment will be trained and provided with a set of protocols, including phone call guidelines and information about the study. Potential subjects who meet the criteria will be invited to participate in the study. Those who agree will sign a consent form. The random assignment schedule, generated using the Research Randomizer software [19], will be compiled by a team member who is not involved in subject recruitment. The assignment of groups will be put in sealed envelopes and revealed sequentially at the time of randomization. The research assistant, after successfully recruiting a subject, will call the researcher responsible for the random assignment. That research team member, who has no knowledge of the identity of the subject, will make the assignment based on a computer number ('1' = mHealth+I group; '2' = mHealth group; '3' = control group).

Interventions

There will be three groups involved in this study, as follows.

mHealth with interactivity (mHealth+I) group

This group of participants will receive a proactive mHealth with interactivity program, which includes two main elements: 1) the mHealth application designed by the research team with information

technological support by a local telecommunications company, and 2) nurse case management supported by a social service team.

The client will be invited to use the mHealth application. The app offers comprehensive education about the causes and etiology, signs and symptoms, risk factors, prevention, and management of pain, hypertension, and diabetes mellitus. The information will be presented in both pictures and videos. The sizes of the pictures, wording, and diagrams are large enough so that the older adults can clearly see and easily understand. Key features of the app include text entry fields that allow participants to keep notes about their vital signs and pain scores, a checklist that helps participants to indicate the origin of pain, signs and symptoms of pain, hyper- and hypo-tension, and hyper- and hypo-glycaemia, a touch screen interface that enables the older adults to fast-scrolling and pinch-to-zoom on the surface, and a reminder function. A reminder message will pop up on the screen of the smartphone when the participant has not used it for more than a week. The participants will be encouraged to read the self-care information that is featured in the app. There is a button for a client-initiated call if they would like to consult a nurse.

The research team has formulated a computerized decision support model based on the guidelines of Hospital Authority and Department of Health, and the comments of an expert panel that includes experienced medical practitioners and advanced practicing nurses to guide an alarming system. When abnormal readings or signs such as hypertensive crisis, hemoptysis and hemiplegia are reported in the client entry, an alert will be sent to the nurse. At the back end, the nurse can also review the client entry. In response to the alert, the nurse will make a call to the client to provide rapid assessment and management according to set protocols. The protocols for each problem involve three components: nurse advice on self-management, referral to social services, and referral to the next level of care. They were developed according to the Omaha System [20] and guidelines from the National Institute for Health and Care Excellence [21].

Besides these client-initiative interactions, the nurse will also provide eight proactive calls (in the first month, a weekly call; in the second and third months, a biweekly call) to the client over the duration of the program, which lasts for three months. A previous study has shown that three months is an adequate dose to bring about change, and it is appropriate to have a more intensive loading dose in the first month and then a maintenance dose in the subsequent two months [22].

In each telephone call, the nurse will: 1) assess the health problems encountered by participants and follow up on contract goals set in previous interactions if appropriate; 2) provide advice on health management and health education, and reinforce self-care behavior; 3) help and empower

participants to set goals and develop their own plans to manage their health problems; and 4) make referrals to community services or the next level of care if appropriate.

The health-social partnership between nurses and social workers is underpinned by Gittell's relational coordination theory in order to achieve better teamwork and a better team climate [23]. Interventions guided by the theory have included frequent and regular meetings and the nurse case manager as team leader. A bimonthly case conference will be held among the nurse, social worker and research team. The multidisciplinary case conference is a way to ensure collaborative and coordinated care for older adults, since it enables the different inter-disciplinary professionals to discuss care options and service provision issues. Issues such as progress and concern about the cases, suggestions for interventions, continued monitoring, requirements for the modification or adjustment of interventions, outcomes and revised plans will be discussed during the meeting. The shared responsibilities are based on standardized protocols and agreed referral forms and records. The community center will provide a trained volunteer to help subjects download the apps and provide technical support, and the center staff will help subjects to complete the process.

mHealth group

The mHealth group will have access to the health content on the mHealth platform. This group will enjoy the same content and client-initiated help if needed. As with the above group, the client is invited to use the mHealth application. The key features of the app will be included. A reminder message will pop up on the screen of the smartphone when participants have not used it for more than one week. The participants are encouraged to read the self-care information that is featured in the app. There is a button for a client-initiated call if they would like to consult a nurse.

Control group

All groups will receive usual community services. The study district provides community-based health talks and basic health checks, such as measuring blood pressure and blood glucose, which are accessible to all residents. Participation is voluntary. Both health and social services are available in the community for those who need them, including referrals for further help if appropriate. These services are, however, episodic and not designed for continuity of care. No mHealth application will

be provided to the participants in this group, but the individuals are free to do their own surfing for e-health information.

The key features of the interventions for the three groups are summarized in table 1.

Data collection

Data will be collected at three time points—at baseline pre-intervention (T1), at three months (T2) when the program is completed, and at six months (T3) to test the sustained intervention effect. The baseline and follow-up data will be collected in both the database of the app (for the mHealth and mHealth+I groups) and the community center (for all groups). A research assistant who is blind to the grouping will be responsible for data collection. The research assistant will be trained and tested on inter- and intra-rater reliability.

Outcome measures

There are four sets of measures: demographics, self-management, and client and health service utilization outcomes.

Primary outcome

The primary outcome is the self-efficacy of the older adults. Self-efficacy will be measured by the General Self-Efficacy Scale. This scale is used to determine how people judge their ability to handle difficult situations or solve their own problems, and thus can be a way to assess the effectiveness of empowerment programs. The 10 items in the Chinese version are rated on a 4-point Likert scale ranging from 1= not at all true to 4= exactly true. Scores on the scale are summated and higher scores indicate greater self-efficacy. The scale has been validated among 655 Chinese adults presenting with high reliability (α=0.89) [24].

Secondary outcomes

The secondary outcomes include self-management outcomes (pain score, blood pressure, capillary blood glucose), client outcomes (quality of life, depression), and health service utilization outcomes (institutionalization and health service utilization [general practitioner, outpatient clinic, emergency room, and hospital admission]).

The pain score will be measured on a visual analogue scale installed in the app. Pain scores will range from 0 (no pain) to 10 (severe pain). The score will be automatically input in the database once the participant has completed the scale in the app. Blood pressure measurement will be performed after 10 minutes of sitting at rest. A standard electronic sphygmomanometer will be used to measure supine blood pressure on the right arm of each candidate (unless contraindicated). Capillary blood glucose level will be measured by a standard capillary glucose meter. Levels greater than 6 mmol/L at fasting or 8 mmol/L at 2 hours or more post-prandial will be considered hyperglycaemic readings [25].

Quality of life will be measured by SF-12v2, which has been translated, validated and proven reliable for use in the Hong Kong Chinese population. The 12 items in the questionnaire were rated on Likert-type scales and summed to provide easily interpretable scales for physical and mental health components. Higher scores indicated better quality of life. The internal consistency and test-retest reliabilities were good (range 0.67-0.82) for all except three scales, and the SF-12v2 summary scores explained >80% of the total variances of the SF-36v2 summary scores [26]. Depression will be measured by the Chinese version of the Geriatric Depression Scale. The scale consists of 15 questions that explore the participants' feelings with dichotomous answers. The scores from each item are summed up. The maximum score is 15, with higher scores representing higher severity of depressive symptoms. Good validity and reliability have been reported in this scale, with criterion-related validity 0.95 and test-retest reliability 0.85 among the older Chinese population [27]. Sensitivity and specificity were 96.3% and 87.5% respectively for a cut-off point of 8.

Institutionalization is defined as the change of living place from an individual's home to an old age or nursing home. Health service utilization will be measured by the number of times the participant attends a general practitioner clinic, a government out-patient clinic (GOPC), the number of unscheduled visits to the emergency department, and hospital admissions. The information can be extracted either from the hospital's clinical management system or by subjective reports from participants. The details can be found in table 2.

Background demographic data

The demographic data, age, gender, marital status, education, work status, years of using a smartphone, accommodation, financial status, family members living in the same household, caretaking support, and the experience of using smartphones or apps will be collected at baseline. The entire set of baseline measures has been validated, and its reliability was confirmed in a previous study [22].

Sample size

The sample size calculation is based on power analysis [28]. Assuming a two-tailed alpha of 0.05, a probability of 0.2 for beta error (80% power), and an effect size of 0.18 after calculating with respect to the same primary outcome measure (self-efficacy) from the results of previous research studies that provided aging-in-place programs with the support of health-social partnerships to older adults [22], 78 participants are required per group. With reference to the 10% to 15% attrition reported in previous programs for community-dwelling older adults, we assume a 20% drop-out rate in this study, thus the total sample size needed is 94 participants per group, i.e. a total of 282 participants.

Data processing and analysis

The data from the coded questionnaire items will be entered twice by the research assistant and the research team member independently into the SPSS software (version 25.0, IBM). Any discrepancies in the data entry between the two will be resolved by retrieving the raw data from the questionnaires. Each of the variables in the dataset will be screened by descriptive statistics to detect potential outliers.

In this study, the Generalized Estimating Equation (GEE) will be adopted to determine the changes or differences between the mHealth+I group, the mHealth group and the control group (between-group effects), the within-group (time) effects, and the interaction effects (group X time). Since there are three time points in this study, the exchangeable working correlation matrix will be used to highlight the same spacing between repeated measurements for each subject [29]. Intention-to-treat (ITT) will be used as the primary analysis in this study. Per-protocol (PP) analysis will be adopted as the secondary analysis. A significant result is indicated when the p-value (level of significance) is less than 0.05 for a two-tailed test.

Discussion/Conclusion

The aging population is increasing worldwide. It is well known that mHealth applications can help older adults to better manage their health in the community and enable high quality health care. However, the trade-off of using this emerging reactive mHealth technology to replace reciprocal face-to-face communication with healthcare professionals is that it may not meet the individual needs of older adults, leading to poor quality of life and increases in health service utilization. The lack of assistance in responding appropriately to the assessment tool in the apps, uncertainty as to when to seek help from healthcare professionals, and older adults' lack of ability and understanding to fully explain their symptoms or chief complaints in the apps further complicate its efficiency in promoting aging in place.

Our study uses a proactive mHealth with interactivity program to help community-dwelling older adults proactively identify and deal with their physical and psychosocial needs, encourage and empower their self-care behavior, and recognize their difficulties in adopting the apps in daily living. Older adults can benefit from supported self-care, equipping them with sufficient knowledge, skills and the confidence to lead a relatively independent life at home. To the knowledge of the research team, this study is the first to adopt a proactive technological approach with the support of a health-social partnership in order to enable older adults to continue living safely and independently in their own community.

Although the strengths of the program have been identified, we also anticipate difficulties during implementation. Due to their poor economic status, some of the older adults may not have Wi-Fi at home. However, the community center and telecommunications company have agreed to provide free Wi-Fi in the center, and a number of SIM cards given to the subjects may facilitate their use of applications. In addition, they may have difficulties using the applications for the first time. Trained volunteers can demonstrate the functions and use of applications in the center whenever the subjects need help.

mHealth apps are valued because of their ability to deliver readily available health content and facilitate e-communication among older adults and healthcare professionals. Older adults can also stay connected with their healthcare providers across temporal and geographical boundaries through the interactive functions of apps. The findings of the study can support policy makers and healthcare professionals in service design and delivery. This model of an interactive mHealth program with the

support of a health-social partnership program, if effective, can be sustained in the study district as well as being introduced and further tested in other districts.

Statements

Acknowledgement

We would like to thank all the community centers for their collaboration with the research team. We would also like to express our gratitude to the telecommunications company for its technical support.

Statement of Ethics

The present study was approved by the ethic committee of the university (reference no: HSEARS20190312002). Written informed consent will be obtained from the participants before commencement of the program.

Disclosure Statement

All authors have no conflicts of interest.

Funding Sources

Not applicable.

Author Contributions

AKCW and FKYW had the initial idea and developed the original study plan. KKPC supported the development and implementation of the interventions. AKCW is responsible for developing the content of the protocols and organizing training workshops. All research sites will be involved in data collection and conduct the study. All authors critically revised the draft manuscript and approved the final manuscript.

References

1. Pew Research Center. Mobile fact sheet; 2019. Https://www.pewresearch.org/internet/fact-sheet/mobile/. Accessed 17 Feb 2020.
2. Census and Statistics Department. Information technology usage: Persons aged 10 and over who had smartphone by age group and sex; 2019. https://www.censtatd.gov.hk/hkstat/sub/gender/itu/index.jsp. Accessed 17 Feb 2020.
3. Teh RC, Mahajan ND, Visvanathan RP, Wilson AP. Clinical effectiveness and attitudes and beliefs of health professionals towards the use of health technology in fall prevention among older adults. International Journal of Evidence-Based Healthcare. 2015;13:213-223.
4. European Commission. Living healthy, ageing well; 2013.
5. mHealth: New horizons for health through mobile technologies: Second global survey on eHealth. WHO Global Observatory for eHealth; 2011.
6. Belisario MJS, Huckvale K, Greenfield G, Car J, Gunn LH. Smartphone and tablet self-management apps for asthma (Review). Cochrane Database of Systematic Reviews. 2013;11:CD010013.
7. Talboom-Kamp PWA, Verdijk NA, Kasteleyn MJ, Harmans LM, Talboom JSH, can den Akker IL, et al. The effect of integration of self-management web platforms on health status in chronic obstructive pulmonary disease management in primary care (e-Vita study): Interrupted time series design. Journal of Medical Internet Research. 2017;19(8):e291.
8. Damant J, Knapp M, Freddolino P, Lombard D. Effects of digital engagement on the quality of life of older people. Health and Social Care in the Community. 2017;25(6):1679-1703.
9. Blomberg K, Wengstrom Y, Sundberg K, Browall M, Isaksson AK, Nyman MH, et al. Symptoms and self-care strategies during and six months after radiotherapy for prostate cancer—Scoping the perspectives of patients, professionals and literature. European Journal of Oncology Nursing. 2016;21:139-145.
10. Goransson C, Eriksson I, Ziegert K, Wengstrom Y, Langius-Eklof A, Brovall M, et al. Testing an app for reporting health concerns—Experiences from older people and home care nurses. International Journal of Older People Nursing. 2018;13:e12181.

11. Chen YRR, Schulz PJ. The effect of information communication technology interventions on reducing social isolation in the elderly: A systematic review. Journal of Medical Internet Research. 2016;18(1):e18.

12. Fathi JT, Modin HE, Scott JD. Nurses advancing telehealth services in the era of healthcare reform. The Online Journal of Issues in Nursing. 2017;22(2):2.

13. De Jong CC, Ros WJG, van Leeuwen M, Schrijvers G. How professionals share an E-Care plan for the elderly in primary care: Evaluating the use of an E-Communication tool by different combinations of professionals. Journal of Medical Internet Research. 2016;18(11):e304.

14. Bodenheimer T. Coordinating care—a perilous journey through the health care system. The New England Journal of Medicine. 2008;358(10):1064-1071.

15. National Health Service. Personalised Health and Care 2020: Using data and technology to transform outcomes for patients and citizens; 2014.

16. Samples C, Zhao N, Shaw RJ. Nursing and mHealth. International Journal of Nursing Sciences. 2014;1(4):330-333.

17. Wong AKC, Wong FKY, Yeung WF, Chang K. The effect of complex interventions on supporting self-care among community-dwelling older adults: A systematic review and meta-analysis. Age and Ageing. 2018;47(2):185-193.

18. Chan AW, Tetzlaff JM, Gotzsche PC, et al. SPIRIT 2013 explanation and elaboration: guidance for protocols of clinical trials. BMJ. 2013;346:e7586.

19. Urbaniak GC, Plous S. Research randomizer (version 3.0). Computer software; 2007. http://www.randomizer.org/

20. Martin KS. The Omaha system: a key to practice, documentation, and information management. 2nd ed. Omaha, NE: Health Connections Press; 2005.

21. National Institute for Health and Care Excellence. Home care: delivering personal care and practical support to older people living in their own homes. NICE guideline; 2015. https://www.nice.org.uk/guidance/ng21/resources/home-care-delivering-personal-care-and-practical-support-to-older-people-living-in-their-own-homes-pdf-1837326858181. Accessed 4 Mar 2020.

22. Wong AKC, Wong, FKY, Chang K. Effectiveness of a community-based self-care promoting program for community-dwelling older adults: A randomized controlled trial. Age Ageing. 2019;48:852-858.

23. Gittell JH. Relationships between service providers and their impact on customers. Journal of Service Research. 2002;4(4):299-311.

24. Leung DYP, Leung AYM. Factor structure and gender invariance of the Chinese General Self-efficacy Scale among soon-to-be-aged adults. Journal of Advanced Nursing. 2011;67(6):1383-1392.

25. Wong SN, Lai KPL, Chow KL. A review on the clinical impact of point of care capillary blood glucose measurement in diabetes patients in public primary care clinics in Hong Kong. Diabetes Manag. 2016;6:45-50.

26. Lam CLK, Wong CKH, Lam ETP, Lo YYC, Huang WW. Population norm of Chinese (HK) SF-12 health survey version 2 of Chinese adults in Hong Kong. Hong Kong Practitioner. 2010;32(2):77-86.

27. Chi I, Yip PSF, Chiu HFK, Chou KL, Chan KS, Kwan CW, et al. Prevalence of depression and its correlates in Hong Kong’s Chinese older adults. The American Journal of Geriatric Psychiatry. 2005;13(5):409-416.

28. Faul F, Erdfelder E, Buchner A, Lang AG. Statistical power analyses using G*Power 3.1: Tests for correlation and regression analyses. Behavior Research Methods. 2009;41:1149-1160.

29. Owusu-Darko I, Adu IK, Frempong NK. Application of generalized estimating equation (GEE) model on students’ academic performance. Applied Mathematical Sciences. 2014;8(68):3359-3374.
