# Supplementary material for: Effect of a Mobile Health Application With Nurse Support on Quality of Life Among Community-Dwelling Older Adults in Hong Kong: A Randomized Clinical Trial
Source: JAMA Netw Open. 2022 Nov 9;5(11):e2241137. doi: 10.1001/jamanetworkopen.2022.41137 (PMC9647479; doi:10.1001/jamanetworkopen.2022.41137)
Supplement: Supplement 2. — Data Sharing Statement [file jamanetwopen-e2241137-s002.pdf]

Data Sharing Statement

Wong. Effect of a Mobile Health Application With Nurse Support on Quality of Life Among Community-Dwelling Older Adults in Hong Kong. *JAMA Netw Open*. Published November 09, 2022. doi:10.1001/jamanetworkopen.2022.41137

Data
Data available: No

Additional Information
Explanation for why data not available: The data that support the findings of this study are available from the corresponding author upon reasonable request.
